# Integrated multi-omic analysis of low-grade ovarian serous carcinoma collected from short and long-term survivors

**DOI:** 10.1186/s12967-022-03820-x

**Published:** 2022-12-17

**Authors:** Kwong-Kwok Wong, Nicholas W. Bateman, Chun Wai Ng, Yvonne T. M. Tsang, Charlotte S. Sun, Joseph Celestino, Tri V. Nguyen, Anais Malpica, R. Tyler Hillman, Jianhua Zhang, P. Andrew Futreal, Christine Rojas, Kelly A. Conrads, Brian L. Hood, Clifton L. Dalgard, Matthew D. Wilkerson, Neil T. Phippen, Thomas P. Conrads, George L. Maxwell, Anil K. Sood, David M. Gershenson

**Affiliations:** 1 Department of Gynecologic Oncology and Reproductive Medicine, The University of Texas MD Anderson Cancer Center, Room T4-3900, Clinical Research Building, 1515 Holcombe Boulevard, Houston, TX 77030 USA; 2 Department of Pathology, The University of Texas MD Anderson Cancer Center, Houston, TX USA; 3 Genomic Medicine, The University of Texas MD Anderson Cancer Center, Houston, TX USA; 4 Women’s Health Integrated Research Center at Inova Health System, Women’s Service Line, Inova Fairfax Medical Campus, Falls Church, VA USA; 5 Gynecologic Cancer Center of Excellence, Department of Obstetrics and Gynecology, Uniformed Services University and Walter Reed National Military Medical Center, Bethesda, MD USA; 6 Department of Anatomy, Physiology and Genetics and Center for Military Precision Health, Uniformed Services University of the Health Sciences, Bethesda, MD USA; 7 Henry M. Jackson Foundation for Advancement of Military Medicine, Inc., Bethesda, MD USA

**Keywords:** Low-grade serous ovarian cancer, Whole-genome sequencing, Global proteomics, Global phosphoproteomics, RNAseq

## Abstract

**Background:**

Low-grade serous ovarian cancer (LGSOC) is a rare disease that occurs more frequently in younger women than those with high-grade disease. The current treatment is suboptimal and a better understanding of the molecular pathogenesis of this disease is required. In this study, we compared the proteogenomic analyses of LGSOCs from short- and long-term survivors (defined as < 40 and > 60 months, respectively). Our goal was to identify novel mutations, proteins, and mRNA transcripts that are dysregulated in LGSOC, particularly in short-term survivors.

**Methods:**

Initially, targeted sequencing of 409 cancer-related genes was performed on 22 LGSOC and 6 serous borderline ovarian tumor samples. Subsequently, whole-genome sequencing analysis was performed on 14 LGSOC samples (7 long-term survivors and 7 short-term survivors) with matched normal tissue samples. RNA sequencing (RNA-seq), quantitative proteomics, and phosphoproteomic analyses were also performed.

**Results:**

We identified single-nucleotide variants (SNVs) (range: 5688–14,833 per sample), insertion and deletion variants (indels) (range: 880–1065), and regions with copy number variants (CNVs) (range: 62–335) among the 14 LGSOC samples. Among all SNVs and indels, 2637 mutation sites were found in the exonic regions. The allele frequencies of the detected variants were low (median12%). The identified recurrent nonsynonymous missense mutations included *KRAS*, *NRAS*, *EIF1AX*, *UBR5*, and *DNM3 mutations*. Mutations in *DNM3* and *UBR5* have not previously been reported in LGSOC. For the two samples, somatic *DNM3* nonsynonymous missense mutations in the exonic region were validated using Sanger sequencing. The third sample contained two missense mutations in the intronic region of *DNM3*, leading to a frameshift mutation detected in RNA transcripts in the RNA-seq data. Among the 14 LGSOC samples, 7754 proteins and 9733 phosphosites were detected by global proteomic analysis. Some of these proteins and signaling pathways, such as BST1, TBXAS1, MPEG1, HBA1, and phosphorylated ASAP1, are potential therapeutic targets.

**Conclusions:**

This is the first study to use whole-genome sequencing to detect somatic mutations in LGSOCs with matched normal tissues. We detected and validated novel mutations in *DNM3*, which were present in 3 of the 14 samples analyzed. Additionally, we identified novel indels, regions with CNVs, dysregulated mRNA, dysregulated proteins, and phosphosites that are more prevalent in short-term survivors. This integrated proteogenomic analysis can guide research into the pathogenesis and treatment of LGSOC.

**Supplementary Information:**

The online version contains supplementary material available at 10.1186/s12967-022-03820-x.

## Background

Patients with low-grade serous ovarian cancer (LGSOC) are usually diagnosed at a younger age and survive longer than those with high-grade serous ovarian cancer (HGSOC) [1]. In a recent single-institution study, the estimated 5-year survival rates were 62.3% for 33 patients with LGSOC and 43.9% for 241 patients with HGSOC; however, they had similar 10-year survival rates (21.2% vs. 22.7%, respectively) [2]. Unfortunately, most LGSOC patients eventually die of the disease because they are relatively chemoresistant and treatment options are still limited [3, 4]. The most common recurrent mutations identified in LGSOC and its putative precursor, serous borderline ovarian tumor (SBOT), are *BRAF* and *KRAS*. *BRAF* and *KRAS* mutations have been identified in approximately 60% of SBOTs and early stage LGSOCs [5–9]. A recently completed clinical trial of trametinib (GOG0281), which inhibits the activity of MEK, the downstream target of the KRAS/BRAF proteins, produced an objective response rate of 26%. Patients with mutations in *KRAS*, *BRAF,* or *NRAS* in this trial had an increased chance of responding to trametinib [10]. However, other genetic aberrations in the MAPK pathway, or those that can bypass the dependence on *BRAF/KRAS/NRAS* have not been fully explored by multi-omics profiling.

Early exome sequencing analyses of a limited number of LGSOCs have revealed very few recurrent mutations [11, 12]. Besides *BRAF/KRAS/NRAS*, novel recurrent mutations reported in LGSOC include *NF1* (9%, 2/23) [13], *ERBB2* (5%, 3/57, in serous borderline tumors) [12], *USP9X* (11%, 2/19), and *EIF1AX* (15%, 3/19) [12]. Other mutations of interest were noted in *FGFR2*, *MAP2K1*, and *ESR1* [14]. A recent sequencing analysis targeting 127 genes in 71 LGSOCs identified additional recurrent gene mutations, including *MACF1* (11%), *ARID1A* (9%), *NF2* (4%), *DOT1L* (6%), and *ASH1L* (4%) [15]. Another recent targeted sequencing analysis of 215 LGSOCs using various platforms identified additional recurrent mutations such as *PIK3CA*, *ATM*, *CREBBP*, *MUTYH,* and *NOTCH3* [16]. However, none of these studies included corresponding normal DNA to validate whether these mutations were true somatic mutations.

To carry out a more comprehensive molecular characterization of LGSOC, we initially performed targeted sequencing mutation analyses of 409 cancer-related genes in 22 LGSOCs and six SBOTs. Subsequently, whole-genome sequencing (WGS) of LGSOC with corresponding normal DNA, RNA sequencing (RNA-seq), and global proteomic and phosphoproteomic analyses were performed. The goal of this study was to identify novel proteogenomic aberrations in LGSOC, especially those associated with poor survival, that could be potential prognostic markers and therapeutic targets.

## Methods

### Patients and pathological materials

This study was approved by the Institutional Review Board of the University of Texas MD Anderson Cancer Center, and all samples were collected after obtaining written informed consent from patients. We obtained fresh-frozen tumor specimens and blood specimens from the MD Anderson Gynecologic Tumor Bank for 31 patients diagnosed with LGSOC and six patients diagnosed with SBOT. The patient demographics and clinical characteristics are presented in Additional file 2: Table S1. These samples were used for targeted sequencing, WGS, RNA-seq, and quantitative proteomic and phosphoproteomic analyses. Blood samples (lymphocytes) were considered as normal tissues for comparison with tumor tissues. Patients with an overall survival of less than 40 months were defined as short-term survivors, and those with an overall survival of > 60 months were defined as long-term survivors. This is based on an analysis of the US population-based Surveillance, Epidemiology, and End Results (SEER) database, in which patients with solid tumors were clustered into six risk groups that differed in median survival (0.5–16.2 years) and high-risk period of death (2.5–12 years). A high-risk period of death was defined as excess annual mortality compared to the age- and sex-matched control population. After the high-risk period (short-term survivors), the mortality gap between cancer patients and the control population stabilizes (long-term survivors) [17]. Since the reported median overall survival of low-grade serous carcinoma patients is between group 1 (high-risk period, 2.5 years) and group 2 (high-risk period, 6 years), the assumption of a high-risk period of approximately 4 years for low-grade serous carcinoma patients would be somewhat arbitrary but reasonable. For the immunostaining study, a tissue microarray containing LGSOCs from 62 patients was constructed using formalin-fixed paraffin-embedded (FFPE) blocks available from our tumor bank. For the “Inova” cohort, as previously described [18], archival FFPE tumors were selected from Inova Fairfax hospital, and LGSOC patients experiencing ≤ 44.4 months (n = 2) or ≥ 93.3 months (n = 4) were prioritized for downstream analysis.

### DNA extraction, library preparation, and targeted sequencing

DNA was isolated from the blood and frozen tissues using a DNeasy Blood and Tissue Kit (Qiagen, Hilden, Germany). Libraries were made using the Ion AmpliSeq Library Kit 2.0 (Thermo Fisher Scientific, Grand Island, NY, USA). Targeted sequencing of 409 genes was performed with the Ion AmpliSeq Comprehensive Cancer Panel (the gene list is provided in Additional file 2: Table S2). Briefly, four PCRs were performed using primers from the panel, which were provided in four different pools with 10 ng of DNA for each PCR multiplexing reaction. The PCR products were partially digested, ligated to adaptors, amplified for five cycles, and purified using Agencourt AMPure XP beads (Beckman Coulter, Indianapolis, IN, USA). The PCR products were then quantified using an Agilent High Sensitivity DNA Kit and Agilent 2100 Bioanalyzer system (Agilent Technologies, Santa Clara, CA, USA) and pooled together in equimolar quantities. The pooled amplified library was subsequently used as a template for emulsion PCR, a process by which DNA was clonally amplified onto beads (Ion Sphere Particles, ISPs) using an Ion PGM Template OT2 200 Kit and Ion OneTouch 2 instrument (Thermo Fisher Scientific). DNA containing ISPs was enriched using Ion PGM Enrichment Beads (Thermo Fisher Scientific) and Ion OneTouch ES instrument. Sequencing primers and polymerase from the Ion PGM Sequencing 200 Kit v2 (Thermo Fisher Scientific) were added to the enriched DNA-positive ISPs before they were placed in an Ion 318 Chip v2 (Thermo Fisher Scientific) for sequencing on an Ion Personal Genome Machine (PGM; Thermo Fisher Scientific).

### Identification of somatic variants from targeted sequencing

For targeted sequencing of the 409 genes, FASTQ files were imported and analyzed using CLC Genomics Workbench software (version 20), as described previously [5] and aligned to the human genome assembly GRCh38 to detect variants. Somatic variants were obtained by filtering variants from tumor DNA from those detected in the matched normal lymphocyte DNA. Six of the 22 LGSOC samples had no matched normal lymphocyte DNA, and the pooled normal variants from the 16 available matched lymphocyte DNA samples were used to detect somatic mutations. Identified somatic mutations were filtered for (a) a variant read count in the tumor sample of ≥ 2, (b) a variant allele frequency (VAF) of ≥ 0.15 in the tumor sample and 0 in the matched normal sample, and (c) common variants in the population with a frequency threshold of 1% in dbSNP129 [19], 1000 Genomes Project [20], Exome Aggregation Consortium [21], and ESP6500 [21]. Somatic variants were confirmed through visual inspection of sequence alignment in the BAM files using CLC Genomics Workbench software (version 20).

### Whole-genome sequencing

We performed WGS as described in a previous publication by our group [22]. Briefly, genomic DNA extracted from frozen tissues and matched blood samples from 14 patients with LGSOC was quantified using the Quant-iT PicoGreen dsDNA reagent and a kit with a Qubit 3.0 fluorometer (Invitrogen). Sequencing libraries were prepared using the TruSeq DNA PCR-Free Library Prep Kit (Illumina), and sequenced on the Illumina HiSeq X platform using the HiSeq X HD Paired-End Cluster Generation Kit v2 (Illumina).

### WGS data analysis and somatic mutation detection

BAM files were generated by aligning WGS reads to the hg19 human reference genome using the BWA software package [23]. Subsequently, duplicate reads were removed using Picard tools (http://broadinstitute.github.io/picard/) and local realignments were performed using the GATK toolkit [24]. Paired tumor and normal BAM files were then used for somatic variant detection. Somatic point mutations and insertions/deletions were identified using MuTect [25] and Pindel tools [26], respectively. The identified somatic mutations were filtered for (a) a total read count in the tumor sample of ≥ 20, (b) a total read count in the germline (blood DNA) sample of ≥ 10, (c) a variant allele frequency (VAF) of ≥ 0.15 in the tumor sample and 0 in the matched normal sample, and (d) common variants in the population with a frequency threshold of 1% in dbSNP129 [19], 1000 Genomes Project [20], Exome Aggregation Consortium [21], and ESP6500 [21]. Oncoplots were generated using MafTools version 2.12 R [27].

### Copy number variant detection

Copy number variations were predicted using the HMMcopy software package [28]. Circular binary segmentation was used to identify regions with copy losses or gains from the copy number log2 ratios of tumor versus matched normal samples [29]. A log2 ratio <  − 0.4 was considered, copy loss, and a log2 ratio ≥ 0.4 was considered copy gain.

### RNAseq analysis and identification of differentially expressed genes

RNA-seq was performed at the MD Anderson Cancer Center Advanced Technology Genomics Core Laboratory as previously described [22, 30]. Total RNA from the same 14 frozen WGS samples was prepared using an RNeasy Mini Kit (Qiagen). However, two RNA samples had RIN values less than 6 and were not used for RNA-seq analysis. Sequencing libraries were prepared using a KAPA Stranded RNA-Seq Kit (Roche Diagnostics) and sequencing was performed on an Illumina HiSeq 4000 system. FASTQ files from RNA-seq were analyzed using CLC Genomics Workbench (version 20) to identify differentially expressed genes. Reads were mapped to the human reference genome GRCh38 and gene expression was estimated using the expectation–maximization (EM) estimation algorithm and reported as transcripts per million (TPM).

### Specimen preparation and tandem mass spectrometr*y* proteomics

Quantitative proteomic analysis was performed on 14 LGSOC tissue samples as described previously [22]. Quantitative proteomic analysis was also performed on an independent cohort of LGSOC tumors, i.e. the “Inova” cohort, previously described [18]. Briefly, laser microdissection was used to collect the whole tumor (combined cancer and stromal cells), and the samples were subjected to pressure-assisted digestion using a barocycler (2320EXT Pressure BioSciences, Inc.) and heat-stable trypsin (SMART Trypsin; Thermo Fisher Scientific, Inc.). Peptide digestion concentrations were determined using a bicinchoninic acid (BCA) assay, and 50 µg of the total peptide was labelled per tandem mass tag channel (TMTpro 11-plex, Thermo Fisher Scientific, Inc.). Sample multiplexes were separated offline using basic reversed-phase liquid chromatography fractionation on a 1260 Infinity II liquid chromatograph (Agilent) into 96 fractions using a linear gradient of acetonitrile (0.69% min) and concatenated into 36 fractions. Ten percent (by volume) of each fraction was removed using liquid chromatography-tandem mass spectrometry (LC–MS/MS). The remaining 90% (volume) was pooled into 12 fractions for serial phosphopeptide TiO_2_ enrichment followed by iron-immobilized metal ion affinity chromatography (Fe-IMAC). Briefly, the peptide fractions were vacuum-dried, resuspended in TiO_2_ binding/equilibration buffer, and bound to TiO2 affinity spin tips (High-Select TiO_2_ Phosphopeptide Enrichment Kit; Thermo Fisher Scientific). The sample flow-through and washes were reserved for subsequent enrichment using ferric nitrilotriacetic acid (Fe-NTA) affinity chromatography (High-Select Fe-NTA Phosphopeptide Enrichment Kit). Each pooled fraction was resuspended in 100 mM NH_4_HCO_3_ and analyzed by LC–MS/MS using a nanoflow LC system (EASY-nLC 1200, Thermo Fisher Scientific) coupled online with an Orbitrap Fusion Lumos Tribrid mass spectrometer (Thermo Fisher Scientific). Briefly, each sample was loaded into a nanoflow high-performance LC system fitted with a reversed-phase trap column (Acclaim™ PepMap™ 100 C18, 2 cm length, nanoViper Trap column, Thermo Fisher Scientific) and a heated (50 °C) reversed-phase analytical column (Acclaim™ PepMap™ RSLC C18, 2 μm, 100 Å, 75 μm × 500 mm, nanoViper, Thermo Fisher Scientific) connected online to an Orbitrap mass spectrometer. The peptides were eluted using a linear gradient of 2% mobile phase B (95% acetonitrile with 0.1% formic acid) to 32% mobile phase B within 120 min at a constant flow rate of 250 nL/min. High-resolution (R = 60,000 at *m/z* 200) broadband (*m/z* 400–1600) mass spectra (MS) were acquired, from which the top 12 most intense molecular ions in each MS scan were selected for high-energy collisional dissociation (HCD, normalized collision energy of 38%) acquisition in the Orbitrap at high resolution (R = 50,000 at *m/z* 200). The monoisotopic precursor selection mode was set to “Peptide,” and the MS1 peptide molecular ions selected for HCD were restricted to z =  + 2, + 3, and + 4. The radio frequency (RF) lens was set to 30%, and both MS1 and MS2 spectra were collected in the profile mode. Dynamic exclusion (t = 20 s at a mass tolerance of 10 ppm) was used to minimize the redundant selection of peptide molecular ions for HCD. Global protein- and phosphosite-level identifications were generated by searching raw data files with a publicly available, non-redundant human proteome database (Swiss-Prot, Homo sapiens [http://www.uniprot.org/]) using Mascot (Matrix Science), Proteome Discoverer (Thermo Fisher Scientific), and in-house tools with identical parameters, as previously described [22]. Differential analyses of the global proteome and phosphoproteome data were performed using the LIMMA package (version 3.8) [31] in R (version 3.5.2), and pathway analysis was performed using Ingenuity Pathway Analysis (Qiagen) and Metascape Analysis [32] (https://metascape.org).

## Results

### Identification of somatic mutations by targeted sequencing

The sequences from all exons of 409 genes generated with the Ion AmpliSeq Comprehensive Cancer Panel from our 22 LGSOC and six SBOT samples had an average coverage of 115′ for each nucleotide. The SNV and indel variants are presented in Additional file 2: Table S3. A total of 176 somatic variants were identified. Six LGSOCs (LGS102, LGS103, LGS116, LGS117, LGS120, and LGS122) had no matched normal tissues and pooled normal reads were used as surrogate controls for these samples to identify somatic variants. Next, we selected 14 missense mutations with a VAF of at least 25% for validation using Sanger sequencing. Table 1 lists the seven gene mutations validated by Sanger sequencing. However, we were unable to validate the other seven mutations (*CTNNB1, EP300, MET, MLH1, PDGFB, PTCH1,* and *TET2*) (data not shown). Sanger sequencing chromatograms for the validated somatic gene mutations are shown in Additional file 1: Fig. S1. Similar to previous reports [7, 16], *BRAF* (4/6 SBOT, 66.7%) and *KRAS* (3/22 LGSOC, 13.6%) mutations had the highest frequencies in our cohort. Two novel mutations identified in this analysis, *UBR5* (c.935A > C; p.E312A) and *EPHA3* (c.2283G > T; p.K761N), have not been previously reported in LGSOC. However, the same *UBR5* mutation (c.935A > C; p.E312A) was reported as a verified somatic mutation in HGSOC (COSMIC database sample ID: COSS1475074). Patient LGS119 had two detected mutations (*EPHA3* and *ATRX*) in her initial tumor, and these mutations were also detected in tumor samples obtained when the patient had a recurrence 17 months after the initial surgery. Previous studies have found that the *EPHA3* mutation K761N is within a highly conserved kinase domain analogous to *that of FGFR2* (K641) [33] and that this mutant protein is likely to function as an oncoprotein by constitutively activating the downstream kinase pathway [34]. Although *TP53* mutations are rare in LGSOC, we detected and validated a *TP53* mutation (c.1025G > C; p.R342P) in exon 10, in contrast to frequently detected *TP53* mutations in exon 5–8. *BRAF* mutations were detected in 4 SBOTs, and *FBXW7* mutations were detected in another SBOT. Mutations in *FBXW7* have previously been detected in SBOT using whole-exome sequencing [35].

**Table 1:** Validated somatic mutations detected by targeted sequencing of all exons in 409 cancer-related genes for 22 patients with LGSOC and 6 patients with SBOT

Patient ID	Diagnosis	Stage	Vital status at last follow-up^a^	Age at diagnosis (years)	Overall survival or length of follow-up (years)	Mutation(s) detected
LGS101	LGSOC	IIIC	Deceased	54	5.1	BRAF p.V600E
LGS102	LGSOC	IIIC	Alive with disease	51	14.3	KRAS p.G12A
LGS103	LGSOC	IIIC	Alive (NED)	66	8.3	KRAS p.Q61H
LGS104	LGSOC	lllB	Deceased	59	4.5	KRAS p.Q61L
LGS105	LGSOC	IIIC	Deceased	52	4.4	TP53 p.R342P
LGS106	LGSOC	lllC	Deceased	66	6.9	UBR5 p.E312A
LGS107	LGSOC	IIIB	Deceased	56	1.4	
LGS108	LGSOC	lllC	Deceased	72	1.9	
LGS109	LGSOC	lllC	Deceased	26	12.0	
LGS110	LGSOC	lllC	Deceased	59	0.2	
LGS111	LGSOC	IV	Deceased	21	2.1	
LGS112	LGSOC	IIIC	Alive with disease	63	15.2	
LGS113	LGSOC	IIIC	Deceased	41	7.3	
LGS114	LGSOC	IIIC	Deceased	70	1.9	
LGS115	LGSOC	IIIC	Deceased	22	4.3	
LGS116	LGSOC	IIIC	Alive (NED)	73	11.6	
LGS117	LGSOC	IIIC	Alive with disease	45	14.9	
LGS118	LGSOC	IIIC	Deceased	64	11.2	
LGS119	LGSOC	IIIC	Deceased	20	17.1	EPHA3 p.K761N; ATRX p. E481D
LGS120	LGSOC	IIIC	Alive with disease	41	9.4	
LGS121	LGSOC	IIIC	Deceased	40	5.5	
LGS122	LGSOC	IIIA	Deceased	38	8.3	
SBOT101	SBOT	IIC	Alive (NED)	48	6.0	
SBOT102	SBOT	IB	Alive (NED)	49	5.6	BRAF p.V600E
SBOT103	SBOT	IIB	Alive (NED)	48	5.3	BRAF p.V600E
SBOT104	SBOT	IA	Alive (NED)	65	5.4	BRAF p.V600E
SBOT105	SBOT	IIIC	Alive (NED)	19	5.2	BRAF p.V600E
SBOT106	SBOT	IIIC	Deceased	59	6.6	FBXW7 p.R505C

^a^*NED* no evidence of disease

### Whole-genome sequencing

**Fig. 1:**
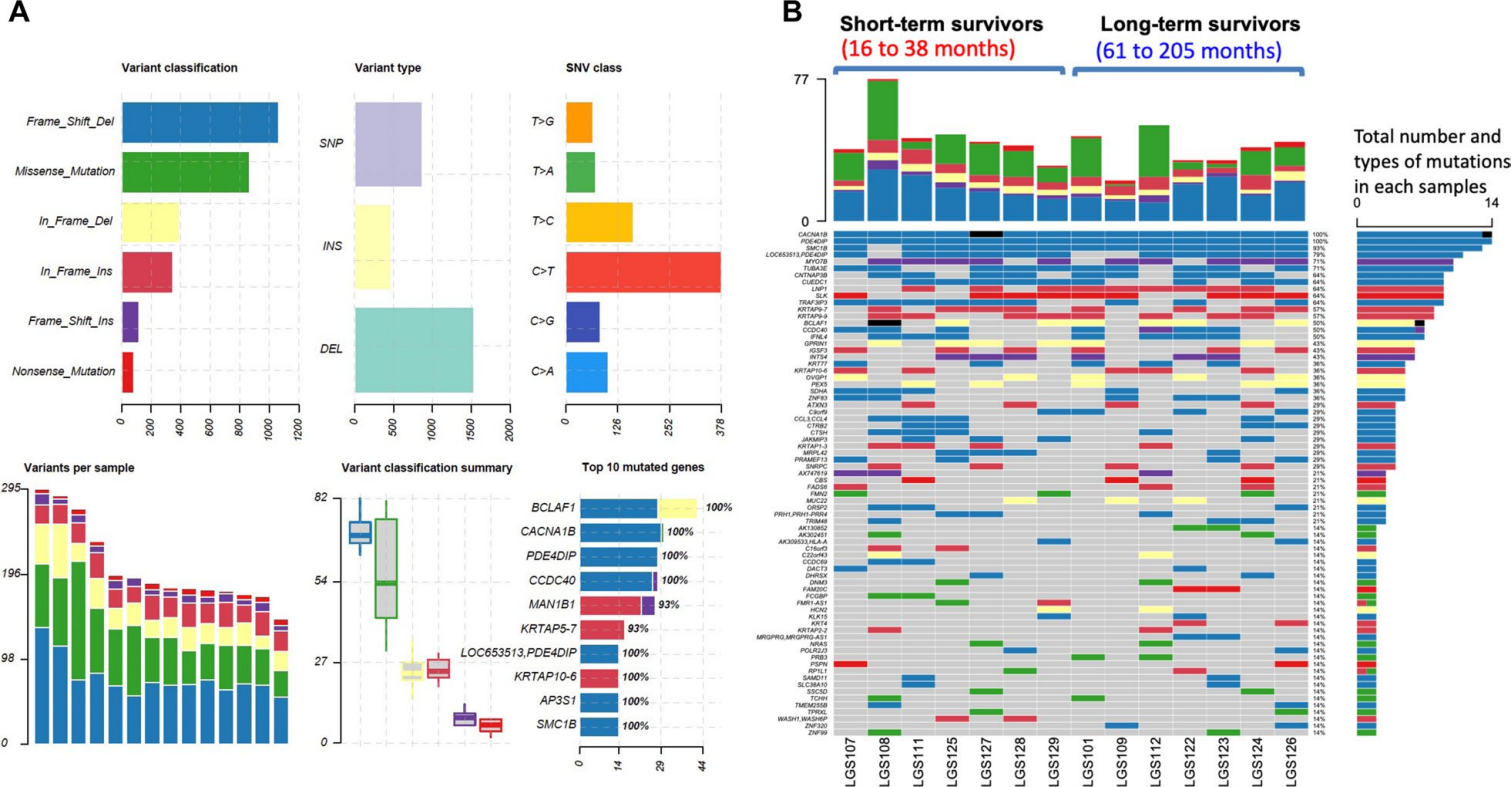
Summary plot of mutational changes. **A** Variant type, SNV class, variants per sample, variant classification summary and the top10 mutated genes detected among all 14 LGSOC samples. Frame_Shift_Del, frameshift deletion; In_Frame_Del, in-frame deletion; In_Frame_Ins, in-frame insertion; Frame_Shift_Ins, frameshift insertion; SNP, single nucleotide polymorphism; INS, insertion; DEL, deletion; SNV, single nucleotide variant. **B** Comparison of total number of mutations and types detected between long-term survivors (n = 7) and short-term survivors (n = 7). Mutations appeared in 2 or more samples were shown. Blue, frameshift deletion; purple, frameshift insertion; yellow, in-frame deletion; red, in-frame insertion; green, missense mutation; orange-red, nonsense mutation

As only a few mutations were detected by sequencing the exons of targeted 409 cancer-related genes, WGS was performed to investigate any single-nucleotide variants, insertions/deletions, copy number changes, or large structural variants on a genomic scale. We retrieved 14 LGSOC samples with matched normal lymphocytes, which was sufficient for WGS, RNA-seq, and quantitative proteomic analyses. DNA extracted from 14 LGSOC tumor tissues with corresponding matched normal lymphocytes was sequenced to a depth of 100′ with the matched blood DNA at a depth of 30′. An average of 2792 million and 936 million sequencing reads were generated for each tumor DNA sample and normal blood DNA sample, respectively. The numbers of SNVs, indels, and regions with CNVs detected for each sample ranged from 5688 to 14,833, 880 to 1065, and 62 to 335, respectively (Additional file 2: Table S4). The calculated tumor mutation burden (TMB) is considered low (2.2–5.3 mutations per Mb) (Additional file 2: Table S4) but is higher than previous reported TMB (0.5–2 mutations per Mb) in LGSOC based on whole exome sequencing [13]. A previous study on colon cancer identified 17 mutations per Mb as the optimal threshold of TMB for predicting MSI status [36]. Thus, these LGSOC are unlikely to be microsatellite instable tumors. Patient LGS126 had the highest number of detected SNVs in the recurrent tumors (n = 14,833). The patient LGS126 had received multiple therapies. Most of the detected variants had a low VAF. There were 546 SNVs or indels within the exon regions, with a VAF ≥ 15% and read counts ≥ 3 (Additional file 2: Table S5). Figure 1A summarizes the types of mutations, variants per sample, SNV classes, and top 10 mutated genes. Frameshift deletion and the transition change C > T were the two most common mutational events in the exonic regions. This finding is similar to that previously reported for cancers [37]. In total, 174 single-nucleotide substitution mutations (nonsynonymous mutations, n = 124; synonymous mutations, n = 50) were identified in the coding regions (Additional file 2: Table S5). Figure 1B compares the total number of mutations and types between long- and short-term survivors. Only mutations that appeared in two or more samples are shown. There was no significant difference in the number of mutational changes between the long-term and short-term survivors (Fig. 1B; Additional file 2: Table S4). The most frequently detected mutations were frameshift deletions or in-frame insertions, such as those in *CACNA1B, PDE4DIP, CCDC40, MAN1B1, KRTAP5-7, SMC1B, AP3S,* and *AP3S1*. A previous whole-exome sequencing analysis of 13 SBOTs and 10 LGSOC identified 396 somatic variants [12], which was less than the number of somatic variants (n = 546) detected in this study. Moreover, the number of indels from the previous exome sequencing (1314/7579; 17.3%) of the 22 LGSOC samples was much lower than that in our study (350/524, 66.7%). This could be because of the different methods used for genome sequencing, different methods for variant calling, and the criteria used for filtering variants. Whole genome sequencing has been shown to be superior to whole exome sequencing for the detection of high-quality coding variants [38].

The tumor DNA purity of each sample was estimated based on the frequency of variants detected in both the tumor and corresponding blood DNA using the CLC Genomics Workbench (Additional file 2: Table S1). By correcting for tumor purity, 2637 somatic variants (missense or indel in exonic regions) with at least three read counts were identified (Additional file 2: Table S6), with VAF ranging from 1 to 100%. Even after adjusting for tumor purity, only 860 somatic variants had a VAF of ≥ 20%. There were 178 nonsynonymous variants in 170 genes, and seven genes had synonymous mutations detected in two or more of the 14 samples. These genes include *KRAS, FCGBP, NRAS, DNM3, SSC5D, TCHH,* and *ZNF99*. Because tumor samples with *DNM3* mutations had higher tumor DNA purity and VAF, we validated these mutations using Sanger sequencing (Additional file 1: Fig. S2). Using Sanger sequencing, we also detected another *UBR5* mutation (c.G953A; p.R318H) in sample LGS129 in addition to the *UBR5* mutation detected by targeted sequencing (Table 1). *The UBR5* mutation (c.G953A; p.R318H) has been previously detected and validated as a somatic mutation in two large intestinal adenocarcinomas (COSMIC sample IDs: COSS1565439 and COSS1650962). Previously reported recurrent mutations in LGSOC, such as *USP9X* and *EIF1AX*, were also detected in two and three of our WGS samples, respectively (Additional file 2: Tables S1, S5, and S6).

**Fig. 2:**
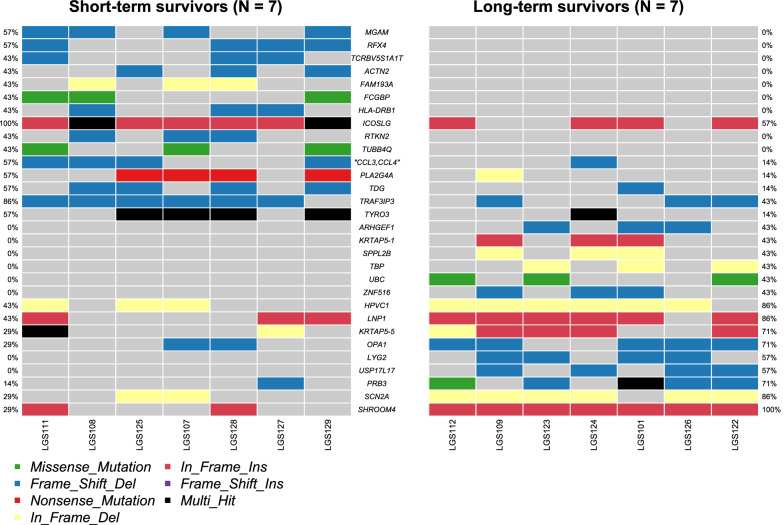
Gene mutations that preferentially occurred in low-grade ovarian serous carcinomas in short-term survivors or long-term survivors

Next, we compared the mutational landscapes of the long- and short-term survivors. Figure 2 shows the 30 gene mutations that were preferentially observed in the long- or short-term survivors. Most mutations were indels, except for four genes (*FCGBP, TUBB4Q, UBC, and PRB3*) with missense mutations. All *the FCGBP* variants were nonsynonymous missense mutations; however, only one of the *TUBB4Q* and *PRB3* variants had a nonsynonymous missense mutation. The remaining variants were synonymous missense mutations, which did not cause amino acid changes.

### Copy number changes in LGSOC

**Fig. 3:**
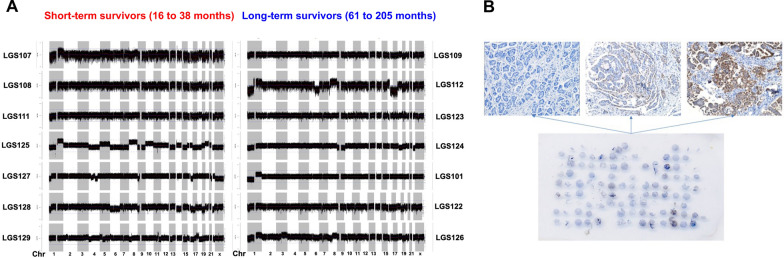
CNVs across all chromosomes from whole-genome sequencing (WGS) data. **A** Comparison of CNVs between short-term and long-term survivors. **B** Immunostaining of p16 protein in LGSOC tissue microarray

Figure 3 compares the CNVs between the long- and short-term survivors across all chromosomes detected in the WGS. Details of the regions with CNVs among the 14 LGSOC samples are listed in Additional file 2: Table S7. Regions with copy number gain or loss in each sample ranged from 62 to 335 in each LGSOC sample, and there was no significant difference in the number of CNVs between the long- and short-term survivors. Chromosomal region (> 100,000 bp) with the most frequent chromosome gain was chromosome 21, and those with the most frequent chromosome loss were chromosomes 1p (n = 6), 6q (n = 5), 9p (n = 6), and 22 (n = 6). These results are similar to those previously reported [6, 39]. As *CDK2NA* is frequently deleted in LGSOC [16], we performed immunostaining for p16 (encoded by *CDK2NA*) on an LGSOC tissue microarray (Fig. 3B). We found that 40 of 62 patient samples had no detectable p16 expression.

### RNAseq analysis

RNA-seq data were generated from 12 LGSOC samples (five long-term and seven short-term survivors) with an average of 66 million reads (range: 54–73 million reads). All patients were initially treated with chemotherapy (taxol plus carboplatin) followed by hormonal therapy, except for one patient, LGS122, who received only hormonal therapies. The gene expression profiles (reported as TPM) of the 12 LGSOC samples are listed in Additional file 2: Table S8. For 117 genes, we observed a differential expression between long- and short-term survivors of > 1.5-fold, with a p-value of ≤ 0.01. Ingenuity pathway analysis indicated that five of these genes (*MKNK1, PPP1R11, PPP2CA, PRKCG,* and *RPS6KA1*) were involved in the canonical ERK/MAPK signaling pathway (Z-score = 2.24; p = 2.28E−03), which was more active in short-term survivors. Table 2 shows a list of differentially associated with potentially targeted drugs. A negative fold-change value indicates higher gene expression in short-term survivors. The greatest difference was observed for *PRKCG*, which was expressed at a 4.82-fold higher level in the short-term survivors. Protein activity of PRKCG can be inhibited by Go6983, a pan–protein kinase C inhibitor. In contrast, *HIF1A* expression was higher in long-term survivors. Overexpression of the associated protein HIF1-alpha has been associated with better survival in early stage squamous cell carcinoma of the oral floor [40]. *SLC1A1* also has higher expression in long-term survivors and is a high-affinity glutamate transporter [41]. However, the role of *SLC1A1* in the development of cancer remains unclear. Upstream regulator analysis indicated that two upstream regulator networks (*BHLHE40* and *HNRNPK*) were activated in long-term survivors compared with short-term survivors. In contrast, three regulator networks (*TCF4*, *USP4,* and *USP9X*) were inhibited in long-term survivors compared with short-term survivors (Table 3).

**Table 2:** Differentially expressed genes between long- and short-term survivors with potential therapeutic targeted drugs

Symbol	Entrez gene name	Fold Change (long/short)	p-value	Short-term mean expression (TPM)	Long-term mean expression (TPM)	Location	Type(s)	Drug(s)
RKCG	Protein kinase C gamma	− 4.82	0.01	3.86	0.77	Cytoplasm	Kinase	Go6983, ingenol mebutate, Ro31-8220
VAMP1	Vesicle associated membrane protein 1	− 1.97	0.00017	15.31	7.77	Cytoplasm	Transporter	Botulinum toxin type B
PER1	Period circadian regulator 1	− 1.82	0.0087	79.62	43.96	Nucleus	Transcription regulator	Avibactam
MKNK1	MAPK interacting serine/threonine kinase 1	− 1.75	0.0081	10.27	5.89	Cytoplasm	Kinase	BAY1143269, dacomitinib, ETC-1907206, QL-X-138, SEL201, tomivosertib
RPS6KA1	Ribosomal protein S6 kinase A1	− 1.7	0.0022	25.9	15.24	Cytoplasm	Kinase	LJH685, PMD-026
POLE	DNA polymerase epsilon, catalytic subunit	− 1.59	0.01	11.9	7.53	Nucleus	Enzyme	Bortezomib/cladribine/rituximabcin interferon
HIF1A	Hypoxia inducible factor 1 subunit alpha	1.96	0.0083	62.38	122.14	Nucleus	Transcription regulator	EZN 2968, PX 478
SLC1A1	Solute carrier family 1 member 1	3.86	0.01	1.18	4.54	Plasma Membrane	Transporter	Riluzole

**Table 3:** Significant upstream regulator networks represented by differentially expressed genes between long-term and short-term survivors with absolute Z-score ≥ 2

Master regulator	Molecule type	Participating regulators	Predicted activation	Activation z-score	p-value of overlap	Target molecules in dataset
BHLHE40	Transcription regulator	BHLHE40	Activated	2.236	0.0129	HIF1A, LPAR1, PER1, PNRC1, SLC7A2
HNRNPK	Other	CEBPB, ERK1/2, HNRNPK, MAP2K1/2, SRC	Activated	2.333	0.00606	CTSV, FOXO1, HIF1A, HMGB2, KLF5, MKNK1, MXD3, NASP, POLE
TCF4	Transcription regulator	TCF4	Inhibited	− 2	0.0333	FOXO1, HIF1A, PPIH, RPS6KA1
USP4	Peptidase	Akt, JINK1/2, P38 MAPK, SMAD4, USP4	Inhibited	− 2.121	0.0119	FOXO1, HIF3A, KLF5, LPAR1, NRP2,PPP1R1B,SMAD3,TPM2
USP9X	Peptidase	ITCH, PRKCB, SMAD4, USP9X, ZAP70	Inhibited	− 2.646	0.00801	ELOVL5, FOXO1, HIF1A, LPAR1, NASP, SMAD3,TPM2

**Fig. 4:**
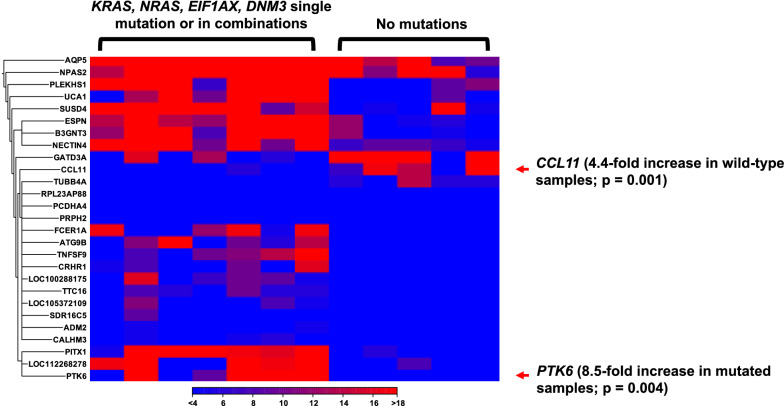
Differentially expressed genes between tumor samples with and without specific mutations (*KRAS, NRAS, EIF1AX,* and *DNM3*)

We also compared the gene expression profiles of LGSOC with and without major recurrent mutations (*KRAS*, *NRAS*, *DNM3*, *and EIF1AX*), and identified a set of differentially expressed genes (Fig. 4). *CCL11* expression was more than fourfold higher in LGSOCs without mutations than in those with mutations. The CCL11 protein can be targeted by bertilimumab. In contrast, *PTK6* expression was more than eightfold higher in LGSOCs with mutations than in those without. PTK6 kinase activity can be targeted by everolimus/vandetanib.

### Global proteomic analysis

**Fig. 5:**
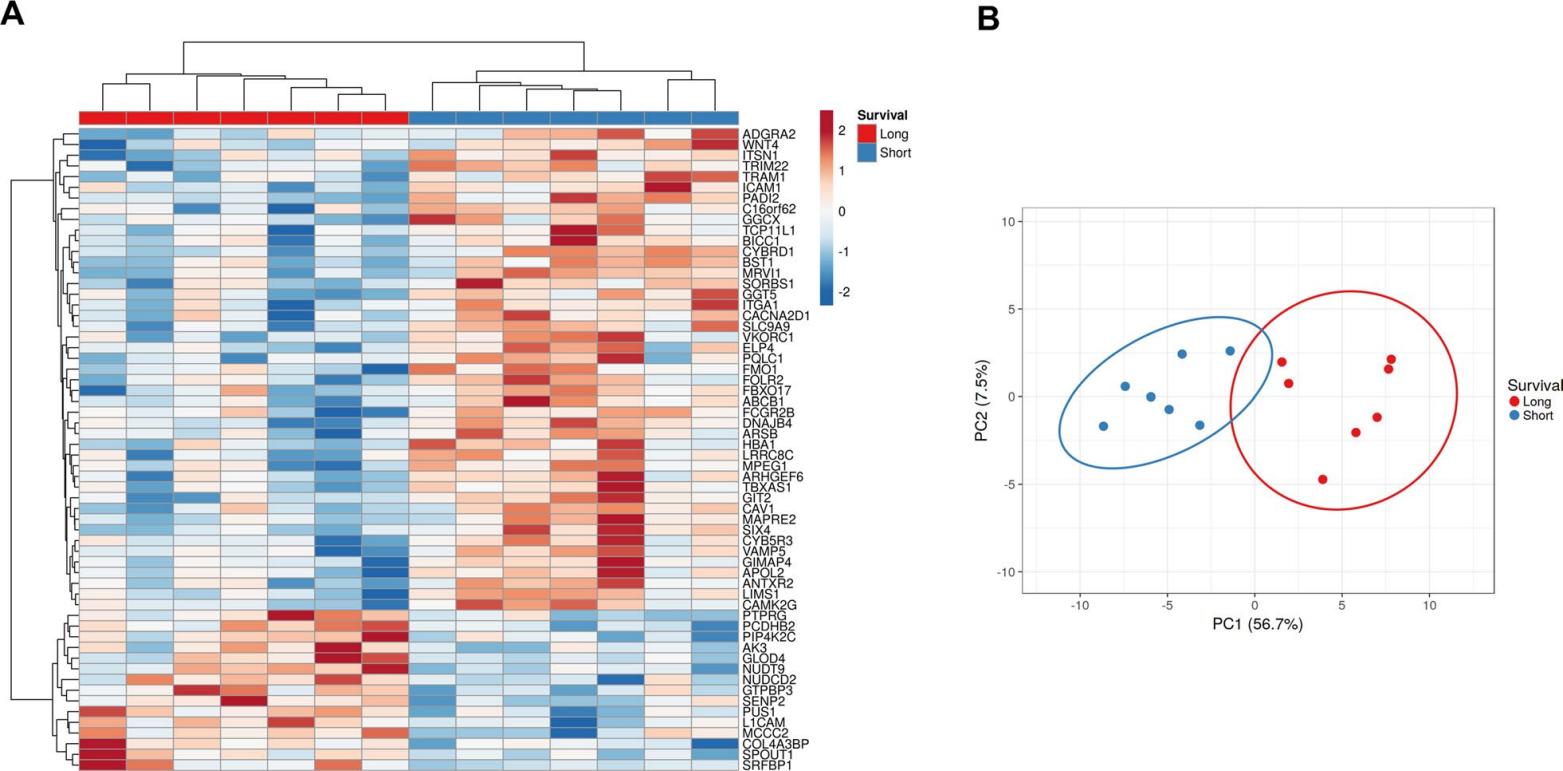
Differentially expressed proteins between long-term and short-term survivors. **A** Heatmap of differentially expressed proteins between long-term and short-term survivors; LIMMA p < 0.01, fold-change ± 1.5. **B** Long-term and short-term survivors were separated by principal component analysis (PCA) with differentially expressed proteins; PCA of protein alterations shown in A served to explain 56.7% and 7.5% of the variance between short and long-term survivors

We measured the expression of 7754 proteins from 14 LGSOC samples (Additional file 2: Table S9). By comparing the proteomic profiles of seven short-term survivors and seven long-term survivors, we identified 60 differentially expressed proteins with a p-value < 0.01, FC ± 1.5 (Fig. 5A, Additional file 2: Table S10). The long-term and short-term survivors were well separated by principal component analysis (Fig. 5B). Several proteins with higher expression in short-term survivors can be targeted by available drugs (Additional file 2: Table S11). Metascape analysis showed that protein alterations were correlated with the enrichment of pathways regulating RNA processing in long-term survivors and intercellular interactions in short-term survivors [32] (Additional file 2: Table S12). Twenty of these differentially expressed proteins were common in both the MDACC discovery and INOVA validation LGSOC samples (Additional file 2: Table S13), with a Spearman’s rank correlation coefficient of rho = 0.48 for protein abundance (Table 4; Additional file 1: Fig. S3). The GTF2F1 and TRIM27 proteins are associated with long-term survival. The transcripts of these two proteins were also associated with a better prognosis based on KMplot analysis [42] (Additional file 1: Fig. S4). HBA1 protein is upregulated in short-term survivors. HBA1 transcript levels also correlated with poor survival (Additional file 1: Fig. S4).

**Table 4:** Twenty co-altered in MDACC discovery and INOVA validation samples, Spearman Rho = 0.48 for protein abundance

Accession	Protein	Gene	MDACC long VS short (Log2 fold-change)	INOVA long VS short (Log2 fold-change)
P02144	MYG_HUMAN Myoglobin	MB	1.28	− 1.11
Q9Y617	SERC_HUMAN Phosphoserine aminotransferase	PSAT1	0.93	1.66
Q969H8	MYDGF_HUMAN Myeloid-derived growth factor	MYDGF	0.83	0.89
Q15645	PCH2_HUMAN Pachytene checkpoint protein 2 homolog	TRIP13	0.66	1.14
Q9UBP6	TRMB_HUMAN tRNA (guanine-N(7)-)-methyltransferase	METTL1	0.56	0.76
Q9Y657	SPIN1_HUMAN Spindlin-1	SPIN1	0.49	0.56
Q9Y4Z0	LSM4_HUMAN U6 snRNA-associated Sm-like protein LSm4	LSM4	0.48	0.79
Q9H0H5	RGAP1_HUMAN Rac GTPase-activating protein 1	RACGAP1	0.48	1.10
Q9ULW3	ABT1_HUMAN Activator of basal transcription 1	ABT1	0.45	0.67
P35269	T2FA_HUMAN General transcription factor IIF subunit 1	GTF2F1	0.41	0.66
Q9UBD5	ORC3_HUMAN Origin recognition complex subunit 3	ORC3	0.40	0.89
Q5T749	KPRP_HUMAN Keratinocyte proline-rich protein	KPRP	0.36	− 1.57
P14373	TRI27_HUMAN Zinc finger protein RFP	TRIM27	0.36	0.86
P25205	MCM3_HUMAN DNA replication licensing factor MCM3	MCM3	0.34	1.20
Q8NBM8	PCYXL_HUMAN Prenylcysteine oxidase-like	PCYOX1L	− 0.54	0.79
O75506	HSBP1_HUMAN Heat shock factor-binding protein 1	HSBP1	− 0.56	− 0.85
P25445	TNR6_HUMAN Tumor necrosis factor receptor superfamily member 6	FAS	− 0.71	− 0.85
Q9BQE5	APOL2_HUMAN Apolipoprotein L2	APOL2	− 0.81	− 0.96
P69905	HBA_HUMAN Hemoglobin subunit alpha	HBA1	− 0.95	− 0.99
P68871	HBB_HUMAN Hemoglobin subunit beta	HBB	− 1.03	− 1.27

### Global phosphosite analysis

For phosphoproteomic analysis, 10,286 phosphosites were quantified in 10 of the 14 LGSOC protein samples. However, 553 of these phosphosites were not registered in the PhosphoSitePlus database (v6.6.0.4; Cell Signaling Technology, https://www.phosphosite.org). The expression levels of 9733 registered phosphosites in the 1327 proteins are provided in Additional file 2: Table S14. By comparing the phosphosite profiles of five short-term and five long-term survivors, we identified 42 phosphosites of specific proteins (with p < 0.01, FC ± 1.5, present in more than 50% of the samples) that were significantly differentially expressed between LGSOC tumors from long- and short-term survivors (Additional file 1: Fig. S5, Additional file 2: Table S15). In long-term survivors, phosphosite alterations correlated with pathways regulating Rho GTPase signaling. In contrast, for short-term survivors, phosphosite alterations correlated with pathways regulating post-translational modifications and diverse kinase-regulated signaling pathways (Additional file 2: Table S16).

### Correlation of significantly differentially expressed genes with co-quantified proteins

Among the 11 samples that had both RNA-seq and quantitative proteomics data, 7680 proteins and transcripts were co-quantified. Only sixty-two significantly differentially expressed genes between short- and long-term survivors from RNA-seq data were co-quantified at the protein level. In general, the protein and transcript expression ratios of short- and long-term survivors shared similar abundance trends. The correlation plot between the transcript ratios of short-term and long-term survivors and the corresponding proteins had a Spearman Rho of 0.352 with a p-value of 0.005 (Additional file 1: Fig. S6). However, the corresponding expressed proteins were largely not significantly altered between short- and long-term survivors. This could be due to that fact that many proteins were not detected in some of the samples. Moreover, Cross-tissue analysis of gene and protein expression in normal and cancerous tissues has shown that the correlation between mRNA and protein abundance is relatively low [43].

## Discussion

LGSOC is a rare disease, with limited therapeutic options. This is the first report of WGS and global proteomic analyses of this tumor. Using paired normal and tumor DNA from the same patients, we identified and validated a few novel recurrent somatic mutations in LGSOC, in addition to *KRAS*, *BRAF, USP9X, and EIF1AX* mutations that have been identified in previous studies. One of the most frequently mutated genes in our cohort was *DNM3* (3/14, 21%), which encodes dynamin 3. Dynamin 3 is a member of a family of guanosine triphosphate (GTP)-binding proteins associated with microtubules and involved in vesicular transport. DNM3 has been shown to play a tumor-suppressive role in cervical cancer, colon cancer, lung cancer, and hepatocellular carcinoma [44–47]. Mechanistic studies in lung cancer have revealed that DNM3 interacts with growth factor receptor–bound protein 2 (GBR2), thereby interrupting the formation of a complex between tyrosine-protein kinase Met (c-MET), GBR2, and signal transducer and activator of transcription 3 (STAT3), which in turn suppresses STAT3 activation [46]. As a result, the loss of DNM3 function leads to the activation of c-MET and STAT3. This suggests that inhibition of c-MET/STAT3 signaling may be a targeted therapy for LGSOC patients with mutated *DNM3*. *DNM3* was also co-mutated with *NRAS* in 2 of both LGSOC samples. It is possible that *DNM3* mutations promote tumor cell growth through the activation of the c-MET/STAT3 pathway. Thus, tumor cells may become less dependent on *NRAS*-activating mutations. It has been shown in melanoma cells that upregulation of c-MET could reduce the dependence on MAPK addiction and lead to MAPK inhibitor resistance [48]. The dependence on *NRAS-* or *KRAS*-mutated LGSOC may be bypassed by additional co-mutations, such as *DNM3* and *EIF1AX*. Further functional analyses on the role of *DNM3* in LGSOC pathogenesis are required.

In this study, recurrent *UBR5* mutations were detected in two LGSOC samples. UBR5 is an E3 ubiquitin ligase that is essential for embryonic development [49]. A previous study showed that high expression of *UBR5* is associated with worse prognosis in ovarian cancer [50]. Tumor-derived UBR5 promotes ovarian cancer growth and metastasis by inducing immunosuppressive macrophages [51]. In contrast, *UBR5* is recurrently mutated in mantle cell lymphoma [52]. Whether the two UBR5 mutated proteins that we detected were functional requires further investigation.

In the current and previous studies, loss of 9p and homozygous deletions of the *CDKN2A/2B* locus are common [12, 15, 16]. We further validated the frequent downregulation of p16 protein expression in LGSOC using tissue microarray. A previous in vitro study showed that ovarian cancer cell lines with p16 loss but with intact pRB were more sensitive to CDK4/6 inhibitors [53]. This observation has implications for the current clinical trial of letrozole plus a CDK4/6 inhibitor (ribociclib) in LGSOC (NCT03673124), in that LGSOC may be more responsive to CDK4/6 inhibition when pRB is intact.

From RNA-seq analysis, we identified several differentially expressed genes (with potential targeted therapeutic drugs) between short-term and long-term survivors. The *USP9X* upstream regulator gene network was activated in short-term survivors (Table 4). *USP9X* is frequently mutated and linked to the mTOR pathway [12, 15]. Short-term survivors with an activated *USP9X* gene network may be candidates for therapeutic interventions targeting mTOR. In addition, we identified differentially expressed genes between LGSOC with recurrent mutations and LGSOC without recurrent mutations (Fig. 4). *CCL11* is highly expressed in short-term survivors and can be bound by a fully human neutralizing monoclonal antibody, bertilimumab [54]. CCL11 is a cytokine that induces MEK-1, ERK1/2, and STAT3 phosphoproteins as a mechanism for conferring anti-apoptotic and cisplatin-resistance potential in ovarian carcinoma [55].

By comparing proteogenomic differences between short- and long-term survivors, we identified proteins associated with short-term survivors. Proteins such as TBXAS1 and BST1 can also be targeted by the currently available drugs. In the phosphoproteomic analysis, 42 phosphosites were significantly altered between the long-term and short-term survivors. The kinases upstream of these phosphosites are potential targets. Phosphorylated ASAP1 was detected at levels more than fourfold higher in short-term than in long-term survivors. One of the upstream kinases that phosphorylate ASAP1 is Src [56]. Targeting Src could be a potential therapeutic intervention for LGSOC with a high expression of phosphorylated ASAP1. Multiple phosphorylated proteins involved in MAPK pathways were also detected but were expressed at different levels in all LGSOC samples. These proteins include RAF1, BRAF, MAPK1, MAPK3, NF1, and other mitogen-activated protein kinase kinases (Additional file 2: Table S11).

## Conclusions

This is the first study to use WGS of LGSOCs with matched normal tissue to detect somatic mutations. We detected and validated novel recurrent mutations in *DNM3* and *UBR5* that have not been previously reported. In addition, we identified novel indels, CNV regions, dysregulated proteins, and phosphosites that were more prevalent in short- and long-term survivors. These proteogenomic data can guide future research into the pathogenesis and treatment of LGSOC.

## Supplementary Information

**Additional file 1: Fig. S1**. Validation of novel somatic mutations from targeted NGS sequencing by Sanger Sequencing. **A** Somatic *UBR5* mutation detected in sample LGS106. **B** Somatic *TP53* mutation detected in sample LGS105. **C** Somatic *ATRX* and *EPHA3* mutations were detected in sample LGS119. **Fig. S2**. Validation of DNM3 somatic nonsynonymous mutations in two LGSOCs by Sanger Sequencing. **Fig. S3**. Twenty differentially expressed proteins shared between two different cohorts (LGSOC_MDACC_DISOVERY and LGSOC_INOVA_VALIDATION). LGSOC_MDACC: n = 7 long (median = 146 months), n = 7 short (median = 24 months; 531 protein alterations, LIMMA p < 0.05). LGSOC_INOVA: n = 4 long (median = 102 months), n = 2 short (median = 33 months), 294 protein alterations, LIMMA p < 0.05). 20 co-altered, Spearman rho = 0.48 for protein abundance; all alterations trends are concordant except for 3 proteins. **Fig. S4**. Protein expression associated with patients’ survival. Up-regulation of GTF2F1 and TRIM27 transcripts correlate with better survival. Up-regulation of HBA1 correlates with poor survival. Figures were generated at KMplot website (https://kmplot.com/analysis/index.php?p=service&cancer=ovar). **Fig. S5**. Differentially expressed phosphosites between long-term and short-term survivors. **A** Heatmap of differentially expressed phosphoproteins between long-term and short-term survivors; LIMMA p < 0.01, fold-change ± 1.5. **B** Long-term and short-term survivors were separated by principal component analysis (PCA) with differentially expressed phosphoproteins PCA of protein alterations shown in A served to explain 59.1% and 10.4% of the variance between short and long-term survivors. **Fig. S6**. Correlation of 62 significantly differentially expressed genes with co-quantified proteins.

**Additional file 2: Table S1**. Patient characteristics and total number of  LGSOC samples with each type of omics analyses performed. **Table S2**. List of 409 genes in the Ion AmpliSeq Comprehensive Cancer Panel assessed by targeted sequencing. **Table S3**. SNVs and InDels identified by targeted sequencing. **Table S4**. Whole-genome sequencing coverage data and variants detected. **Table S5**. Summary of missense and InDel somatic mutations in exon regions with variant allele frequency > 15%. **Table S6**. All missense and InDel somatic mutations in exon regions with variant allele read count ≥ 3. **Table S7**. Regions with CNVs detected in each sample. **Table S8**. Expression values from RNAseq analysis of 12 LGSOC samples. **Table S9**. Global proteomic data. **Table S10**. Differentially expressed proteins between short-term and long-term survivors. **Table S11**. Proteins with higher expression in short-term survivors with targeted drugs. **Table S12**. Enrichment of pathways correlated with differentially expressed Protein between long-term and short-term survivors by Metascape analysis. **Table S13**. Characteristics of LGSOC_Inova Validation Samples. **Table S14**. Global phosphosites detected. **Table S15**. List of 42 phosphosites of specific proteins that were significantly differentially expressed between LGSOC tumors from long-term survivors and those from short-term survivors. **Table S16**. Enrichment of pathways correlated with differentially expressed phosphosites in Protein between long-term and short-term survivors by Metascape analysis.

## Data Availability

The datasets used in this study are available upon request from the corresponding author. All the data analyzed in this study are included in this published article and its supplementary information files. The whole genome sequencing BAM files have been deposited at the European Genome-phenome Archive (EGA), which is hosted by the EBI and the CRG, under accession number EGAD00001009626.

## Abbreviations

LGSOC: Low-grade serous ovarian carcinoma
WGS: Whole-genome sequencing
CNV: Copy number variant
Indel: Insertion and deletion variant
