# Supplementary material for: Integrated multi-omic analysis of low-grade ovarian serous carcinoma collected from short and long-term survivors
Source: J Transl Med. 2022 Dec 17;20:606. doi: 10.1186/s12967-022-03820-x (PMC9758924; doi:10.1186/s12967-022-03820-x)

Fig. S1. Validation of novel somatic mutations from targeted NGS sequencing by Sanger Sequencing

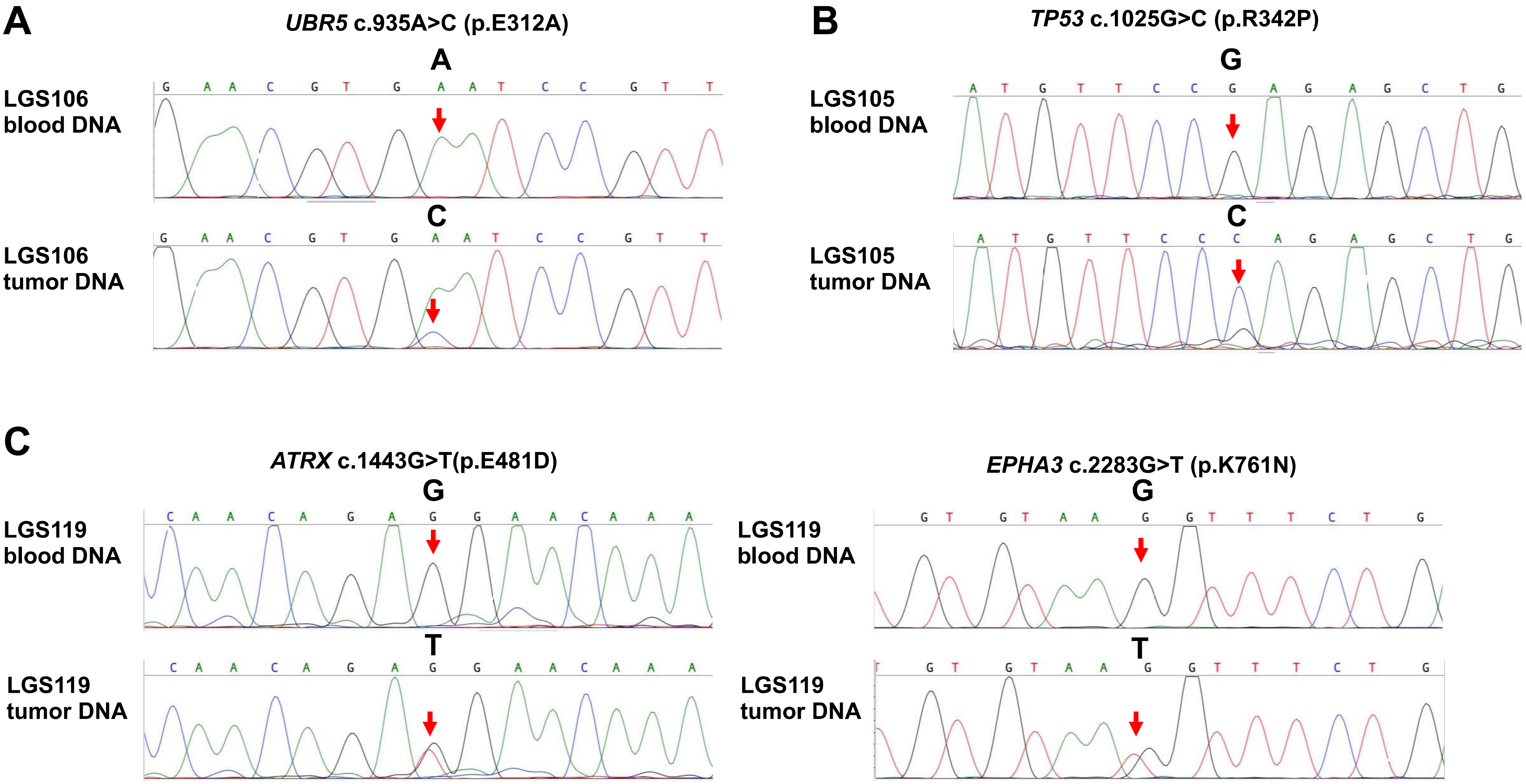

Fig. S2. Validation of *DNM3* somatic missense nonsynonymous mutations in two LGSOCs by Sanger Sequencing

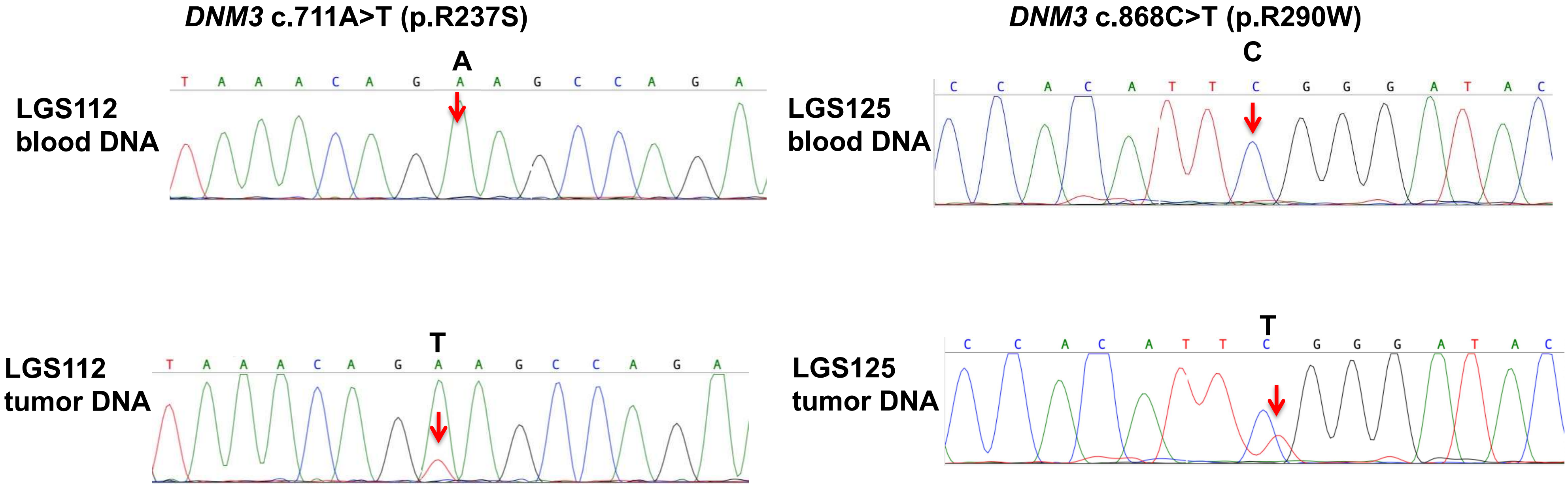

Fig. S3. Twenty differentially expressed proteins shared between two different cohorts (LGSOC_MDACC_DISOVERY and LGSOC_INOVA_VALIDATION). LGSOC_MDACC: n = 7 long (median = 146 months), n = 7 short (median = 24 months; 531 protein alterations, LIMMA $p < 0.05$). LGSOC_INOVA: n = 4 long (median = 102 months), n = 2 short (median = 33 months), 294 protein alterations, LIMMA $p < 0.05$). 20 co-altered, Spearman rho = 0.48 for protein abundance; all alterations trends are concordant except for 3 proteins.

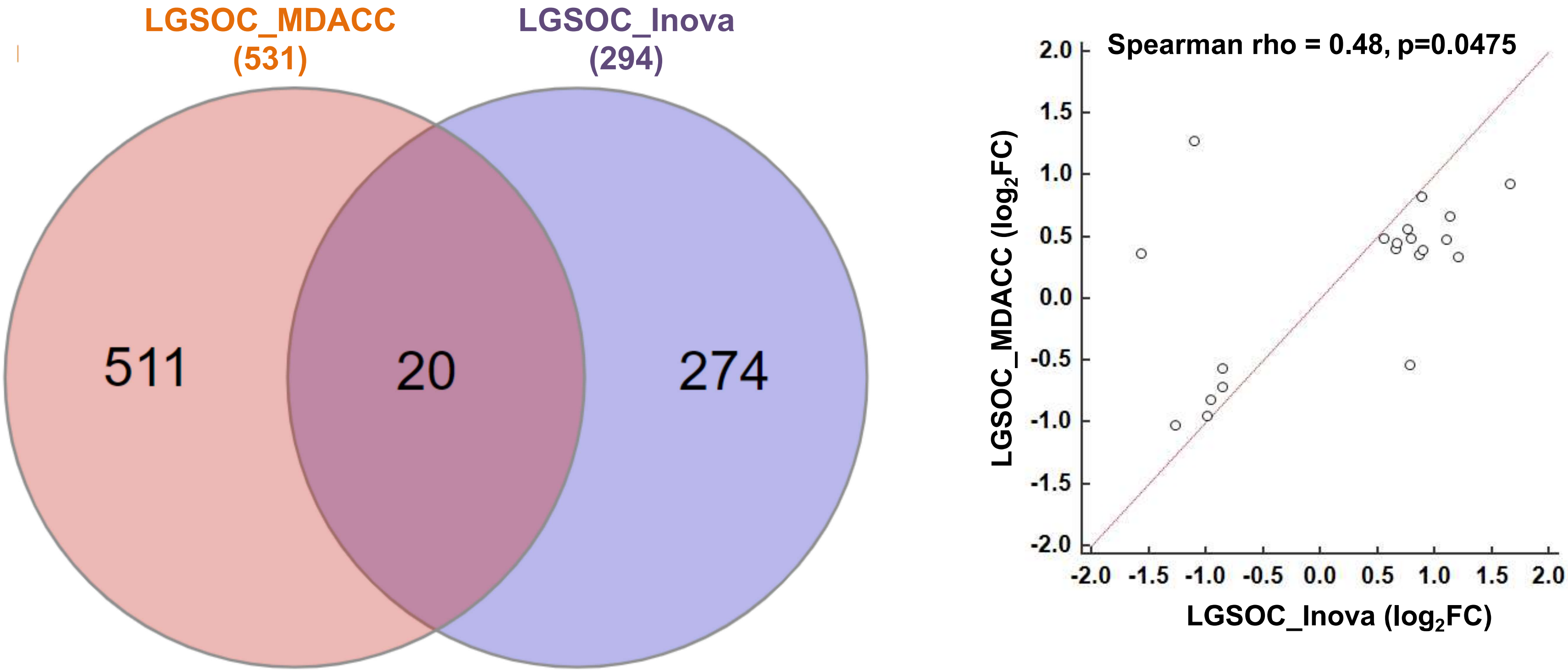

Fig. S4. Protein expression associated with patients' survival. Up-regulation of GTF2F1 and TRIM27 transcripts correlate with better survival. Up-regulation of HBA1 correlates with poor survival. Figures were generated at KMplot website (https://kmplot.com/analysis/index.php?p=service&cancer=ovar)

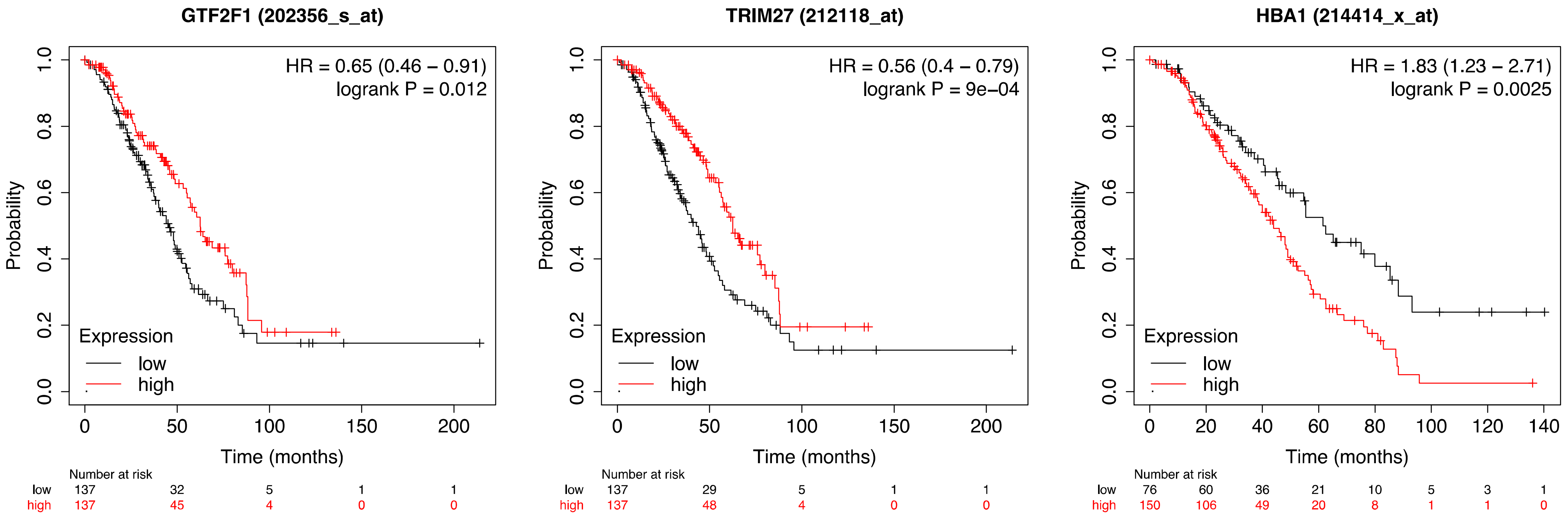

Fig. S5. Differentially expressed phosphosites between long-term and short-term survivors. **A** Heatmap of differentially expressed phosphoproteins between long-term and short-term survivors; LIMMA $p < 0.01$, fold-change ± 1.5. **B** Long-term and short-term survivors were separated by principal component analysis (PCA) with differentially expressed phosphoproteins PCA of protein alterations shown in A served to explain 59.1% and 10.4% of the variance between short and long-term survivors.

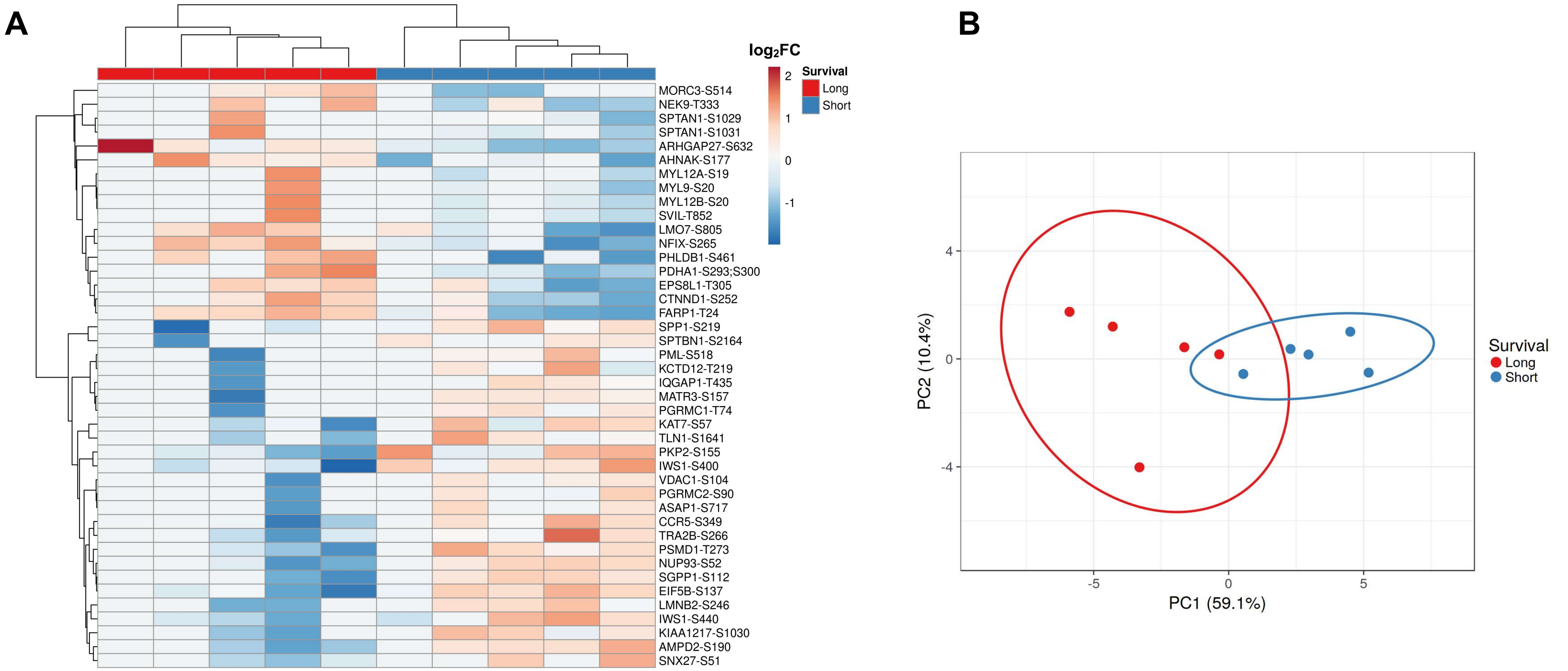

Fig. S6. Correlation of 62 significantly differentially expressed genes between long and short-term survivors with co-quantified proteins. logFC, log base 2 fold change of transcript and the corresponding protein between long and short-term surivors.

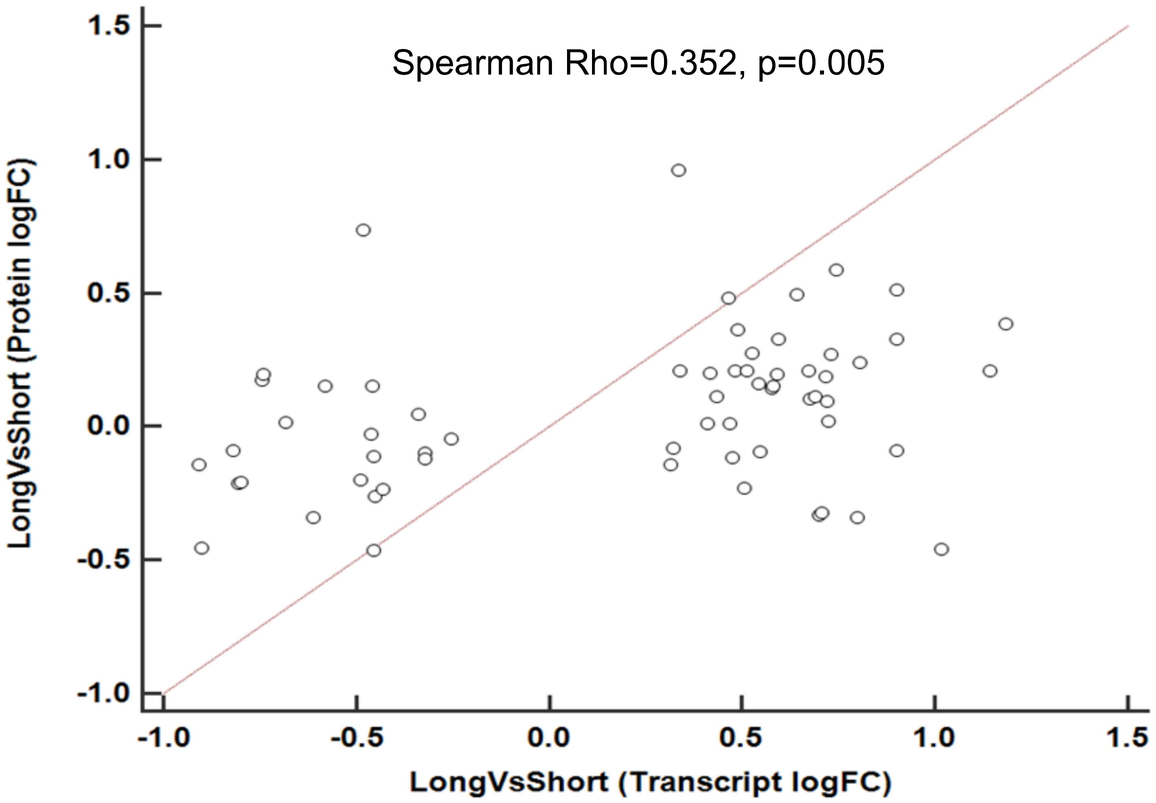

Supplement: Supplementary file 1 — Additional file 1: Fig. S1. Validation of novel somatic mutations from targeted NGS sequencing by Sanger Sequencing. A Somatic UBR5 mutation detected in sample LGS106. B Somatic TP53 mutation detected in sample LGS105. C Somatic ATRX and EPHA3 mutations were detected in sample LGS119. Fig. S2. Validation of DNM3 somatic nonsynonymous mutations in two LGSOCs by Sanger Sequencing. Fig. S3. Twenty differentially expressed proteins shared between two different cohorts (LGSOC_MDACC_DISOVERY and LGSOC_INOVA_VALIDATION). LGSOC_MDACC: n = 7 long (median = 146 months), n = 7 short (median = 24 months; 531 protein alterations, LIMMA p < 0.05). LGSOC_INOVA: n = 4 long (median = 102 months), n = 2 short (median = 33 months), 294 protein alterations, LIMMA p < 0.05). 20 co-altered, Spearman rho = 0.48 for protein abundance; all alterations trends are concordant except for 3 proteins. Fig. S4. Protein expression associated with patients’ survival. Up-regulation of GTF2F1 and TRIM27 transcripts correlate with better survival. Up-regulation of HBA1 correlates with poor survival. Figures were generated at KMplot website (https://kmplot.com/analysis/index.php?p=service&cancer=ovar). Fig. S5. Differentially expressed phosphosites between long-term and short-term survivors. A Heatmap of differentially expressed phosphoproteins between long-term and short-term survivors; LIMMA p < 0.01, fold-change ± 1.5. B Long-term and short-term survivors were separated by principal component analysis (PCA) with differentially expressed phosphoproteins PCA of protein alterations shown in A served to explain 59.1% and 10.4% of the variance between short and long-term survivors. Fig. S6. Correlation of 62 significantly differentially expressed genes with co-quantified proteins. [file 12967_2022_3820_MOESM1_ESM.pdf]
